# A CMOS Time-Resolved Fluorescence Lifetime Analysis Micro-System

**DOI:** 10.3390/s91109255

**Published:** 2009-11-18

**Authors:** Bruce R. Rae, Keith R. Muir, Zheng Gong, Jonathan McKendry, John M. Girkin, Erdan Gu, David Renshaw, Martin D. Dawson, Robert K. Henderson

**Affiliations:** 1 Institute for Integrated Micro and Nano Systems, The School of Engineering, The University of Edinburgh, The King's Buildings, Mayfield Road, Edinburgh, EH9 3JL, UK; E-Mails: Keith.Muir@ed.ac.uk (K.R.M.); Robert.Henderson@ed.ac.uk (R.K.H.); David.Renshaw@ee.ed.ac.uk (D.R.); 2 Institute of Photonics, University of Strathclyde, 106 Rottenrow, Glasgow, G4 0NW, UK; E-Mails: Zheng.Gong@strath.ac.uk (Z.G.); Jonathan.McKendry@strath.ac.uk (J.M.); Erdan.Gu@strath.ac.uk (E.G.); M.Dawson@strath.ac.uk (M.D.D.); 3 Department of Physics, Durham University, South Road, Durham, DH1 3LE, UK; E-Mail: J.M.Girkin@durham.ac.uk

**Keywords:** CMOS, single-photon avalanche diodes, GaN, micro light-emitting diodes (micro-LEDs), fluorescence lifetime, micro-system

## Abstract

We describe a CMOS-based micro-system for time-resolved fluorescence lifetime analysis. It comprises a 16 × 4 array of single-photon avalanche diodes (SPADs) fabricated in 0.35 μm high-voltage CMOS technology with in-pixel time-gated photon counting circuitry and a second device incorporating an 8 × 8 AlInGaN blue micro-pixellated light-emitting diode (micro-LED) array bump-bonded to an equivalent array of LED drivers realized in a standard low-voltage 0.35 μm CMOS technology, capable of producing excitation pulses with a width of 777 ps (FWHM). This system replaces instrumentation based on lasers, photomultiplier tubes, bulk optics and discrete electronics with a PC-based micro-system. Demonstrator lifetime measurements of colloidal quantum dot and Rhodamine samples are presented.

## 1. Introduction

Fluorescence based analysis is a fundamental research technique used in the life sciences. However, conventional fluorescence intensity measurements are prone to misinterpretation due to illumination and fluorophore concentration non-uniformities. Thus, there is a growing interest in time-resolved fluorescence detection, whereby the characteristic fluorescence decay time-constant (or lifetime) in response to an impulse excitation source is measured. The sensitivity of a sample's lifetime properties to the micro-environment provides an extremely powerful analysis tool. However, current fluorescence lifetime analysis equipment tends to be bulky, delicate and expensive, thereby restricting its use to research laboratories. Progress in miniaturization of biological and chemical analysis instrumentation is creating low-cost, robust and portable diagnostic tools capable of high-throughput, with reduced reagent quantities and analysis times. Such devices will enable point-of-care or in-the-field diagnostics. In this paper, we report an integrated fluorescence lifetime analysis system capable of sub-nano second precision with the core of the instrument measuring less than 1 cm^3^, something hitherto impossible with existing approaches. To accomplish this, recent advances in the development of AlInGaN micro-LEDs and high sensitivity CMOS detectors have been exploited [1,2]. CMOS technology is key to both detection and excitation in our system providing compact, low cost, high speed electronic signal -processing circuitry for the photodetectors and vertically integrated drivers for the micro-LEDs. Furthermore, we demonstrate an array of pixellated fluorescence analysis sites with potential for multiplexed, high-throughput sensors, with reduced alignment tolerances. Combined with recent advances in on-chip, real-time lifetime computation [3,4] this work represents as significant step towards practical, micro-scale lifetime sensors, without the need for additional external hardware or sophisticated software post-processing.

## 2. Background

### 2.1. Fluroescence Lifetime

Fluorophores have associated with them an exponential fluorescent decay transient after the removal of the excitation source, which defines their characteristic lifetime [5]. Due to the random nature of fluorescence emission, a fluorescent sample's associated lifetime is the average time the molecules in a sample spend in the excited state before photon emission occurs.

A sample's fluorescence lifetime, τ, is determined by the rate at which the sample leaves the excited state (Equation 1). The transition can occur via two mechanisms, either by fluorescence emission (at rate Γ) or by competing non-radiative processes (represented collectively as K_nt_):

(1) $$\tau =\frac{1}{\Gamma +\Sigma K_{nt}}$$

A fluorophore's quantum yield (Θ) is the ratio of emitted photons to the number of absorbed photons. This can be represented by Equation 2:

(2) $$\Theta =\frac{\Gamma }{\Gamma +\Sigma K_{nt}}$$

For a given excitation light intensity, a fluorophore's brightness (molecular brightness, q) can be calculated if the molecular absorption coefficient (ε) is known, Equation 3:

(3) q=ɛ×Θ

The absorption coefficient of a fluorophore is usually constant; therefore, changes in a fluorophore's brightness can usually be attributed to changes in the sample's quantum efficiency. Therefore, from Equations 2 and 3, if the fluorescence intensity changes this will usually result in a change in sample lifetime. Due to the fact fluorescence intensity is a composite property of a sample, dependent on sample quantity and concentration as well as instrument set-up, it is very sensitive to sample variation and is subject to interference from scattered light. This makes the observation of small intensity changes very difficult. Conversely, fluorescence lifetime is an intrinsic fluorophore property, independent of sample volume and concentration. Lifetime analysis is also less sensitive to instrument setup. Fluorescence lifetime is therefore a more robust analysis method compared to intensity measurement, capable of observing subtle changes in sample conditions [6].

The rate of non-radiative recombination is dictated by the fluorophore's electron structure and its interaction with the environment. Non-radiative decay mechanisms include [7]:

- Inter-system crossing
- Collisional or static quenching
- Solvent effects
- Resonance energy transfer.

Fluorescence intensity is related to lifetime according to Equation 4 (for a mono-exponentially decaying sample). The equation assumes that the sample has been excited by an infinitely sharp (δ-function) light pulse. The time-dependent intensity at time t, I(t), is given by:

(4) $$I(t)=I_{0}\exp(\frac{-t}{\tau })$$

Fluorescence lifetime is independent of fluorophore concentration but dependent on the sample's local environment. Thus, lifetime detection allows precise quantitative data about both fluorophore distribution and local environment to be obtained, while avoiding the problems related to fluorescence intensity imaging such as photo-bleaching [8]. Fluorescence lifetime detection can also be used to differentiate between fluorophores with overlapping spectra, but exhibiting different decay characteristics. Typical fluorescence decay times of organic compounds fall between a few hundreds of picoseconds and several nanoseconds. There are a number of different imaging experiments for which time-resolved detection can be used; these include, multiple fluorophore labeling [9], quantitative detection of ion concentrations and oxygen and energy transfer characteristics using fluorescence resonance energy transfer (FRET) [10].

**Figure 1.**
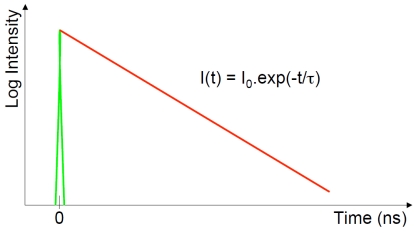
In the time domain, fluorescence intensity decay is measured directly as a function of time.

There are two predominantly used techniques for measuring the fluorescence lifetime of a sample: the frequency-domain and time-domain methods. In the frequency domain a sample is excited by an intensity modulated light source. This results in the fluorescence emission being modulated at the same frequency, but with a phase shift due to the intensity decay law (Equation 4) of the sample [7,11] and a reduction in the modulation depth. In the time domain the intensity decay of a fluorescent sample is directly measured as a function of time, following absorption of a short excitation pulse (Figure 1).

The design and application of bio-chips and micro-devices that can perform analysis for biomedical applications rapidly and inexpensively in a miniaturized environment has been the focus of much research [12,13]. A need for the development of simple, robust, cost-effective medical devices capable of rapidly screening for multiple diseases and to monitor pathogens has been identified as a key step in the fight against infectious diseases, especially in developing areas [14]. The miniaturization of diagnostic devices has the potential to increase throughput and reduce the cost of a wide range of diagnostic tests [15]. Furthermore, micro-scale systems often require reduced reagent quantities, resulting in reduced operating costs. The aim of much research into device miniaturization is to produce a point-of-care device, capable of performing sample analysis quickly and easily at a patient's bed-side or in a doctor's surgery [16,17].

Drug discovery is an area of research that could benefit from high-throughput miniaturized devices [18]. There is also on-going research into the development of implantable in vivo analysis devices [19]. Micro-analytical systems have been developed for the analysis of a wide range of analytes including oxygen [20], glucose, chemical and biological agents [21] as well as fluorophores and biological samples such as DNA [22]. One of the key challenges in the development of such devices is the integration of the different technologies required to produce a functional device. In a fluorescence-based device this would include sample excitation and detection elements alongside a sample handling mechanism such as micro-fluidics [23].

### 2.2. Excitation Sources

Traditionally, fluorescence excitation is achieved using laser sources or mercury or halogen lamps. Fluorescence analysis systems often contain several sources of different wavelength in order to allow samples of different excitation wavelength to be analysed. Arc and incandescent lamps are commonly used excitation light sources due to their broadband continuous emission, but their size, low efficiency and low stability make them unsuitable for miniaturized portable analysis systems. Gas discharge lamps have also been used for fluorescence excitation; these devices operate in a free-running mode and are different to control. Furthermore, the high supply voltage which they require (>5 kV) is difficult to provide in a compact format. Currently, the standard excitation source for time-domain fluorescence lifetime analysis is the pulsed laser diode. Available over the full visible wavelength spectrum these devices provide a low cost solution, relative to the femto-second Ti:Sapphire laser, to pulsed sample excitation. Once placed within a cooling heat sink these devices are therefore significantly larger than devices based on CMOS technologies (which are in the order of a few millimeters squared).

In 1995, Araki and Misawa [24] demonstrated the use of commercially available blue InGaN/AlGaN LEDs for fluorescence lifetime measurements. Driven by an external RLC (resistor, inductor, capacitor) circuit and controlled by an avalanche transistor, these devices generated 4ns wide optical pulses with a 10 kHz repetition rate and a peak optical power of 40mW. In order to operate the avalanche transistor required a 300 V collector voltage. In addition, the inclusion of an inductive component in the drive circuit makes its realization in an integrated microelectronic circuit difficult. Using a high-gain photomultiplier tube and TCSPC hardware, accurate fluorescence lifetime measurements of Quinine-Sulfate are presented using these LED devices as an excitation source. This demonstrated how pulsed LEDs were suitable for consideration as a light source in time-domain fluorescence analysis.

Fluorescence lifetime analysis using micro-LED excitation was demonstrated in [25] 64 × 64 matrix-addressable LED array driven by external hardware with a pulse width of 2 ns was used to excite a sample of rhodamine-123, with the subsequent fluorescence decay being captured by a commercially available photomultiplier tube (PMT). These InGaN/GaN devices measured 20 μm in diameter and were capable of producing 40 nW average optical power with a 4 V bias. Lifetime measurements of rhodamine-123 excited with a blue (460 nm) micro-LED and capture using a fast photomultiplier are presented. Being matrix-addressable the intersection of a row and column signal will activate and array element. As array sizes increases this creates potential fan-out problems. Furthermore, each row signal must supply current to all elements in that row; this limits pulse capabilities due to the associated slow RC time constants. This issue can be addressed by active address logic or by providing each element with a local driver circuit.

The development of low-cost, miniaturized excitation sources for a full optical lab-on-a-chip is often neglected. Several groups have demonstrated fluorescence excitation using vertical cavity semiconductor devices [12,26] where they have been integrated into a micro-analytical device. These devices, however do not allow drive electronics and signal processing circuitry to be included on the same substrate.

### 2.3. Detectors

Photon counting applications require detectors of single-photon sensitivity, these include: micro-channel plate PMTs, high-speed amplified PMTs, discrete photodiodes and avalanche photodiodes. These devices tend to be discrete components, requiring separate power supplies and a communication interface. Furthermore, they tend to be physically large and delicate (especially PMT devices). PMT devices are also sensitive to magnetic fields making difficult their integration into medical devices such as magnetic resonance imagers (MRI). A number of groups have demonstrated micro-scale fluorescence detection using a variety of different detectors. In [27] Patounakis *et al.* demonstrate CMOS detection of fluorescence lifetime decays using conventional CMOS photodiodes and on-chip signal processing circuitry. These devices rely on the integration of photodiode current to estimate photon intensity and does not display single-photon sensitivity.

There has been significant progress in recent years in the development of CMOS image sensors, mainly driven by the demand from the mobile telephone market. Originally developed for the CCD image sensors, the pinned photodiode has now been utilized in CMOS image sensors, offering reduced dark current and transfer noise. In [28] a CMOS image sensor, aimed specifically at fluorescence lifetime imaging, with a 256 × 256 pinned photodiode array is implemented in a 0.18 μm image sensor specific CMOS process. A novel two-stage charge transfer pixel structure allows excitation and background photons to be subtracted from the detected signal leaving only signal due to fluorescence emission. Similar to the work presented in [27], fluorescence decay data is calculated by varying the time at which the photodiode is switched from passing charge to the drain node to storing charge for readout.

Recent developments in the design of CMOS compatible single-photon avalanche diodes [1] allow extremely sensitive detectors to be integrated alongside signal processing circuitry. In order to gather photon arrival time data, from which fluorescence lifetime can be extracted, a number of circuit techniques have been proposed. These include; on-chip time-to-digital converters [29] and in-pixel time-gated counters [30]. Single-photon avalanche diodes offer micro-scale single-photon detection capabilities and their ability to capture fluorescence data has been well-documented [30,31], and [32]. They offer a number of other significant advantages; including being robust devices which are not destroyed by high light levels, insensitive to magnetic fields and are relatively easy to manufacture [33].

Despite growing interest in fully integrated CMOS based SPAD systems, SPAD detectors based on other semiconductor materials have also become more widespread. Despite the inability to integrate electronics on the same substrate as the detection element, these devices are often packaged alongside a second external quenching device [34,35]. The advantage of non-CMOS based devices is that the wavelength sensitivity of the device is no longer constrained by the junction depth and bandgap of silicon and can be tailored to individual applications. This can lead to SPAD detectors capable of detection in the near infra-red [36,37]. Unfortunately, these devices cannot take advantage of the large scale production capabilities and investment that has been made in silicon-based CMOS technology and do not offer a low cost solution to single-photon counting.

### 2.4. Miniaturisation

In [15], a micro-system integrating a GaN thin-film LED alongside a CdS distributed Bragg reflector (DBR) filter, a PDMS microfluidic channel and Si PIN photodetector is presented. As this system was intended for intensity analysis, LED operation is DC and is driven by external hardware. Despite having a silicon substrate, this system includes no signal processing or LED control circuitry. The use of a microfluidic channel allows the sample of interest to be easily introduced into the micro-system. This device employs a planar topology, with the excitation and detection elements located on the same substrate, allowing the micro-fluidic device to be easily placed on top of the system with just 2 mm of separation between the sample and the detector.

Similar work is presented in [25], whereby a VCSEL excitation source emitting at 773 nm has been integrated alongside emission filters and PIN photodetectors. As in [30], this device is intended for fluorescence intensity analysis and the VCSEL light source was not designed for short pulse excitation. Based on III-V materials the inclusion of CMOS electronics in this system is not possible.

## 3. Device Implementation

**Figure 2.**
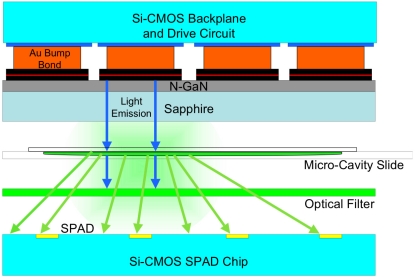
Cross-section of the two-chip micro-system.

In this paper we present a micro-system that incorporates pixellated excitation and detection devices in a two-chip “sandwich” structure (Figure 2). Combining the excitation source with a photodetector, on-chip driving electronics and lifetime signal processing circuitry, our devices represent a highly integrated lab-on-a-chip (LoC) system. Pixellation of detector and emitter arrays at 200 μm pitch are compatible with inkjet-spotted, multiplexed assay formats. The 777 ps optical pulse width is the shortest reported pulse for a CMOS-driven micro-LED device emitting at 450 nm and is suitable for excitation of commonly used, short lifetime fluorophores such as Rhodamine and Fluoroscein. Furthermore, the inclusion of an optical filter reduces measurement error caused by the detection of scattered excitation light.

### 3.1. Excitation Array

**Figure 3.**
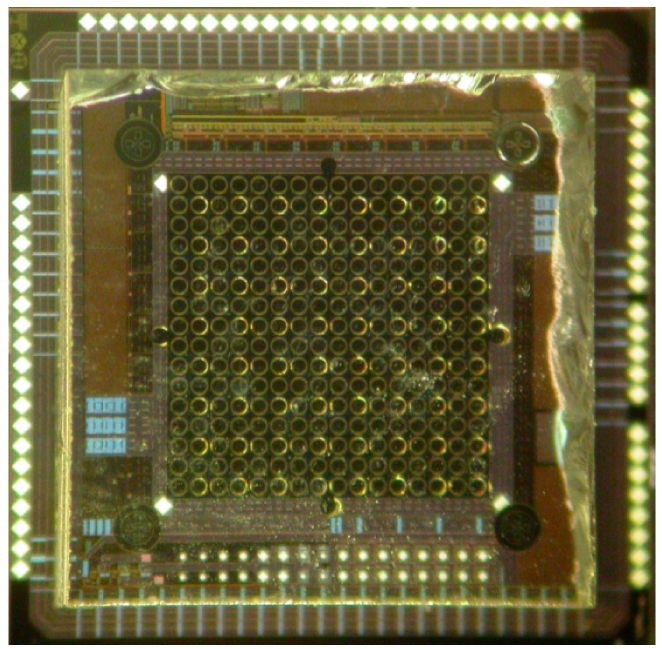
AlInGaN micro-LED array bump-bonded to an 8 × 8 CMOS driver array.

Sample excitation is achieved using an 8 × 8 array of 72 μm diameter AlInGaN blue micro-pixellated light-emitting diodes (micro-LEDs) fabricated from “standard” InGaN/GaN quantum well blue LED wafers (planer n- and p- type GaN layers) grown on *c*-plane sapphire substrates by metal organic chemical vapor deposition [38]. This micro-LED array is bump-bonded to an equivalent array of LED driver circuits realized in a standard low-voltage 0.35 μm CMOS technology (Figure 3). Each array element is individually addressable, with a dedicated driver circuit per micro-LED element. The wavelength spectra of the CMOS driven blue micro-LED device peaks at a wavelength of 450 nm.

**Figure 4.**
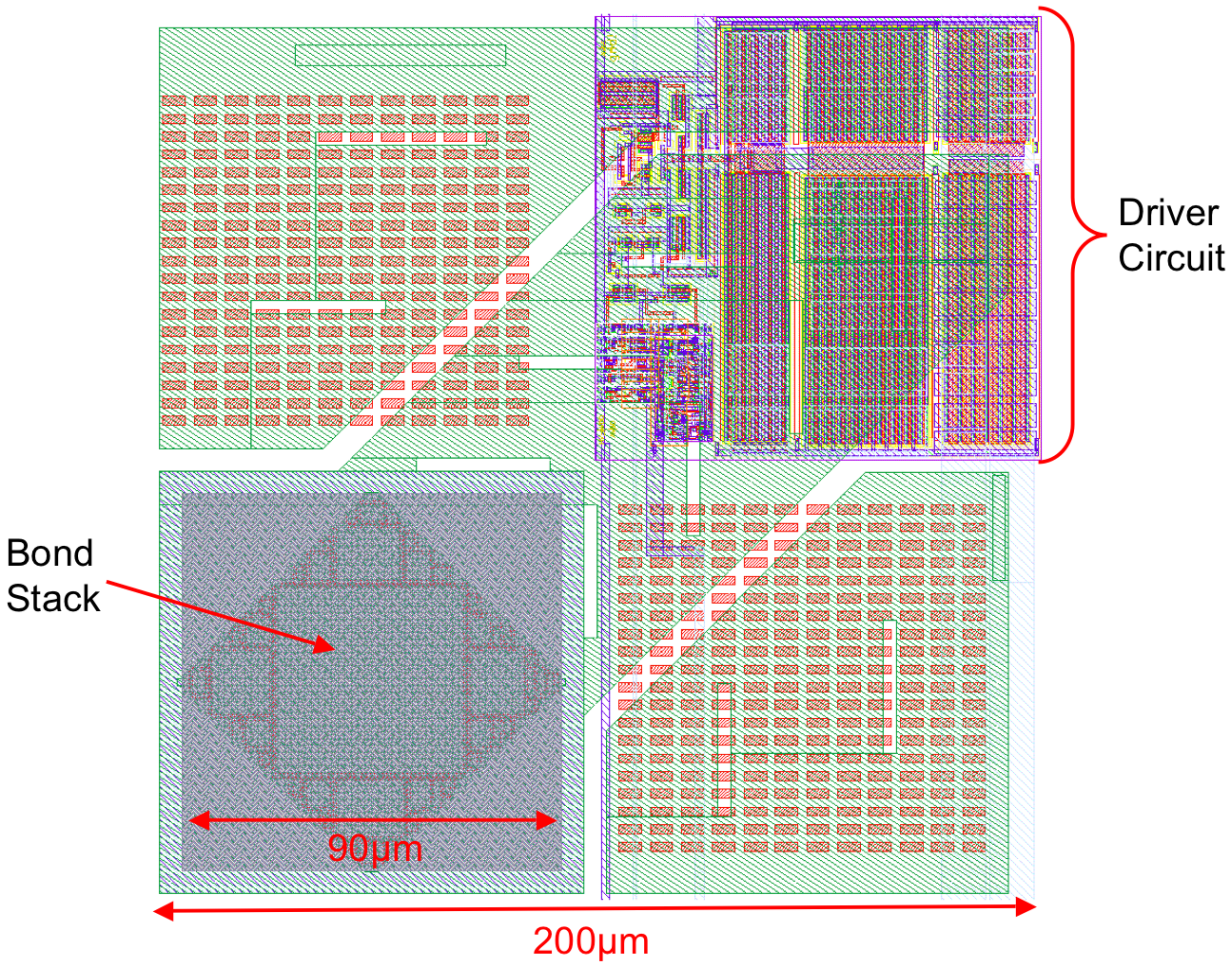
Layout of a single micro-LED driver element.

Each element of the CMOS driver array measures 200 μm × 200 μm with a 200 μm pitch. A pixel contains a dedicated driver circuit, driving a full metal bond-stack to which the micro-LED array was bump-bonded (Figure 4). All driver input signals were based on 3.3 V logic before being level-shifted to a higher user-definable voltage (LED_VDD), to a maximum of 5 V. This allows standard 3.3 V logic to be used for the addressing and control logic in the pixel before the signal level is increased to LED_VDD (requiring the use of physically larger transistors capable of handling 5 V).

**Figure 5.**
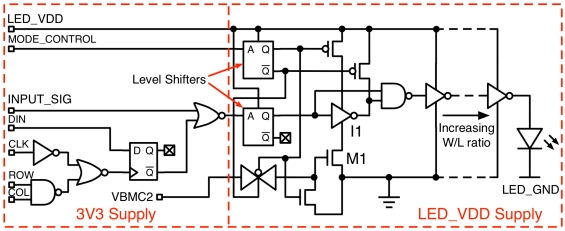
CMOS driver element, illustrating the output buffer and short pulse generation circuitry.

**Figure 6.**
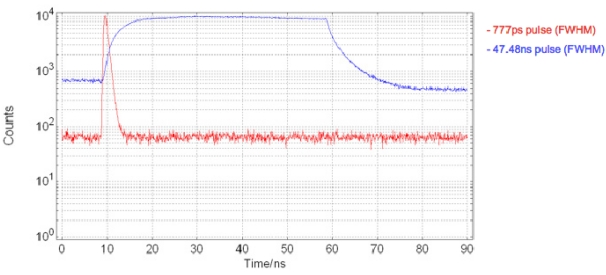
Shortest and longest micro-LED excitation pulses.

The driver circuit (Figure 5) is capable of producing optical pulses of user–definable width variable from 47.48 ns down to 777 ps, FWHM (±180 ps estimated measurement error, based on PMT RMS jitter), Figure 6. By placing a square-wave signal on INPUT_SIG, the delay through inverter I1 defines the pulse width. The inverter delay can be adjusted via the gate voltage (VBMC2) of the current starving NMOS transistor M1. The level-shifted DC, pulsed, or square wave signal is then passed to an output buffer designed using transistors capable of handling up to 5 V. To minimize load capacitance on the input signal while maximizing the drive strength of the circuit, an output buffer comprising a chain of inverters of increasing transistor width/length ratios has been implemented.

An on-chip voltage controlled oscillator (VCO) has also been implemented within the 8 × 8 driver array. This circuit is capable of producing a square wave signal with a tunable frequency range from 7 MHz to 800 MHz. The design features fine and course adjustment of the VCO frequency. The core frequency of the VCO is defined by the number of elements in the ring oscillator and the delay through each of these elements. Current starving transistors are placed within the ring oscillator and the gate voltage of these transistors is defined off-chip, thus allowing fine adjustment of the core ring oscillator frequency. The output of the ring oscillator is then passed to a digital divider circuit capable of dividing the input signal by 0, 4, 16 or 64 and hence producing a course selection of lower frequency signals. The VCO output could be used as the input signal to the drivers of the main array, defining the repetition rate of a square wave or pulsed input signal. By producing a square wave input signal on-chip the need for an off-chip clock (such as a crystal oscillator) has been removed, potentially reducing system size and cost. The performance of the micro-LED excitation array is summarized in Table 1.

### 2.2. Detection Array

**Figure 7.**
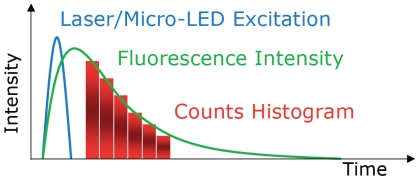
Illustration of a fluorescence lifetime decay captured using a time-gated counter circuit.

A compact micro-system for time-resolved fluorescence was achieved by making use of CMOS technology's ability to integrate signal processing circuitry on the same chip as a sensor array, thereby allowing detector data to be directly processed. We describe how time domain, time-gated fluorescence lifetime analysis has been implemented on a CMOS chip. Using this method, the sample of interest is excited by a pulsed light source. The subsequent lifetime decay is captured within a series of two or more gated count windows. Using the count values obtained in each window a histogram of the fluorescence decay curve can be generated (Figure 7). A fluorescence lifetime is then obtained by applying a lifetime extraction algorithm to the histogram data.

A SPAD detector has been implemented which allows single photon detection through the action of avalanche breakdown in a p+/deep n-tub photodiode, reverse biased above its breakdown voltage (Geiger mode). These are situated in a 16 × 4 array pitch-matched to the micro-LEDs, allowing histogram and lifetime analysis without the need for external photon counting hardware and significantly reducing the amount of data to be broadcast off-chip. Direct observation of SPAD output pulses is also possible from an array of addressable SPADs situated directly within the micro-LEDs for confirmation of the integrated lifetime analysis techniques.

**Figure 8.**
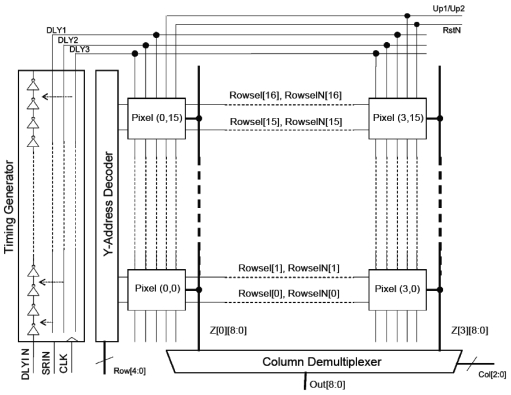
System block diagram.

A CMOS time-resolved analysis system has been designed, consisting of a fully addressable array of 16 × 4 array of SPADs integrated with on-chip signal processing and timing circuits. Each pixel measured 100 μm × 200 μm. The pixels incorporated two 9-bit ripple up-down counters with a novel time-gating mechanism allowing fully programmable scanning of time resolved events over a 48 ns range with a 408 ps resolution. The device was controlled by a FPGA and photon count histograms were captured and displayed by a PC. Figure 8 shows a system block diagram. Both the SPAD counter array and the micro-LED array were based on this architecture. By processing raw SPAD data locally within each pixel, the amount of data that would otherwise have to broadcast across the chip and potentially off-chip is minimized.

**Figure 9.**
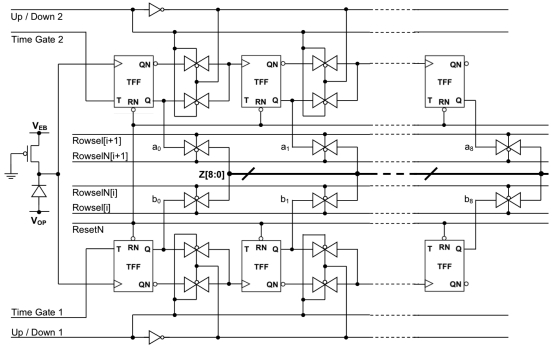
SPAD circuit with passive quench transistor and two associated 9-bit ripple counters.

Within each pixel two 9-bit up/down ripple counter circuits were implemented, these were designed using toggle (T-type) flip-flops (FF). The SPAD pulses provided the asynchronous clock to the first T-type FF in the counter (Figure 9). A ripple counter was chosen to minimize the clock loading, since no synchronous count behaviour is required. An up-down counter was used to allow background light compensation although this was not implemented. Time-gated operation is accomplished by providing the toggle input of the first T-type FF in the counter with short pulses, which are generated within the pixel from delayed versions of the 3.68 MHz system clock broadcast to the array from the on-chip timing generator. The 9-bit word-length of each counter circuit allows 512 counts to be gathered before it is necessary to read-out the counter data. Two counters allow direct on-chip implementation of the two-gate RLD lifetime extraction method. Photon collection efficiency is improved by enabling the counters in immediate succession during the two time gate bins within one clock period.

The timing generator consists of a 120-element tapped delay line composed of current limited buffers. The buffer unit delay is 408 ps with 44 ps RMS jitter at 3.3 V at room temperature. Three delayed versions of the 3.68 MHz system clock are generated; each delayed output can be selected independently under the control of a latched shift register. Time-gate widths can be selected from 408 ps to 48 ns with a resolution of 408 ps. Each element of the delay line consists of a two-inverter buffer with an in-line current starving transistor. The gate bias of the current starving transistor was passed off-chip, allowing the user to control the delay through each element in the delay chain. This allows the user to extend the maximum length of the delay generator at the expense of minimum time-gate width.

**Figure 10.**
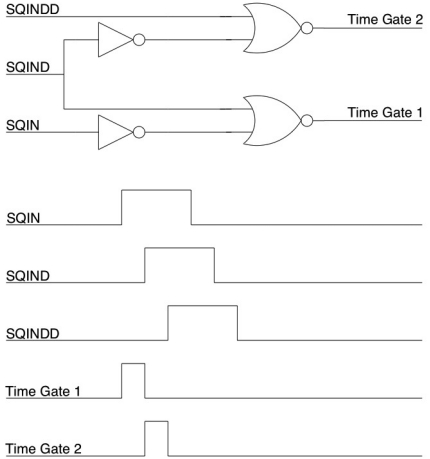
Circuit and timing diagram for in-pixel time-gate generation.

The delay line generates three delayed versions of the system clock. The time delay between these three signals is user definable, by selecting the element of the tapped delay line which outputs the delayed clock. These three delayed clock signals are then broadcast globally across the chip to each pixel in the array. Circuitry within each pixel then generates time gates of width equal to the time delay between the signals. A schematic of the circuitry used to achieve this and a timing diagram of the process is shown Figure 10. By using the difference between two signals broadcast to each pixel via the same route, jitter in the enable signal is minimised, as is the bandwidth requirement of the clock bus drivers. The delay setup by the tapped delay line is user definable via PC control of the FPGA. In this way, the time gates can be easily modified to fit the sample of interest.

## 4. System Configuration

**Figure 11.**
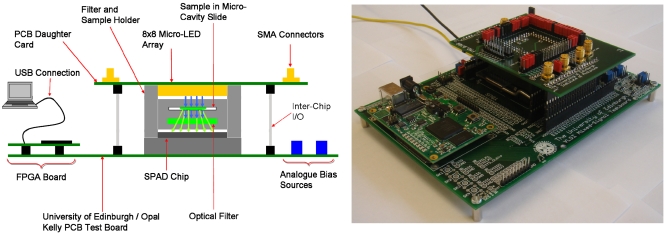
Complete two-chip micro-system. The PCB daughter card is physically supported by the filter and sample holder and stacked header pins.

A dedicated PCB daughter card was designed, with the micro-LED device situated on the under-side of the PCB, facing the SPAD detector chip located on a FPGA test board. Electrical connection to the daughter card was made via stacked header pins. This technique allowed the distance between the micro-LED device and the SPAD detector chip to be adjusted. The excitation and detection arrays have a minimum separation of 3 mm. Both devices shared the same core power supplies and ground connections. These supplies and all other bias supplies, apart from the negative SPAD detector, were generated on the test board PCB and derived from the 5V supply of the USB connection. The negative supply required by the SPAD detector was generated by an external power supply. The devices shared a single FPGA situated on the test board (Opal Kelly, XEM3010), which generated the digital input signals to both devices. An optical filter and the sample of interest were placed between the devices. A plastic holder was designed to house these two elements. This holder provided a light tight enclosure for the packaged SPAD chip, an optical filter, a sample held in a micro-cavity slide and a packaged micro-LED device. Figure 11 shows the configuration of the two-chip system.

**Figure 12.**
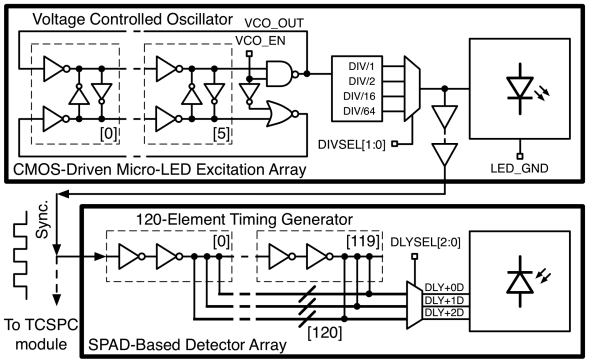
The on-chip VCO provides the square-wave input to the micro-LED driver array and the SPAD time-gate generator circuit.

The output from the on-chip VCO, situated on the micro-LED driver, defines the repetition rate of the LED device. This signal is passed off-chip (to a SMA connector on the daughter card) and is used as the synchronization input to a time-correlated single photon counting (TCSPC) module (Becker and Hickl, SPC-130), or the detector on-chip timing generator circuit (Figure 12). Using this method, the excitation and detection elements of the system can be accurately synchronized. A 514 nm long pass filter (Semrock, LP02-514RU-25) was chosen to separate the excitation light from the fluorescence emission. This allows a range of fluorophores with emission spectra greater than 514 nm to be evaluated while maximizing the rejection of excitation light.

## 4. Results

To assess the sensitivity of the SPAD detector and in-pixel counters, fluorescence lifetime analysis of a series of quantum dot samples of different concentrations was conducted. Quantum dot samples with an emission wavelength of 548 nm were prepared at concentrations of 50, 25, 10, 1, 0.1 and 0.01 μM. 45 μL of each sample was loaded into a single cavity (15 mm diameter) glass microscope slide (Fisher Scientific, UK, MNK-140-010A) and sealed with a 0.12 mm thick cover-slip.

**Figure 13.**
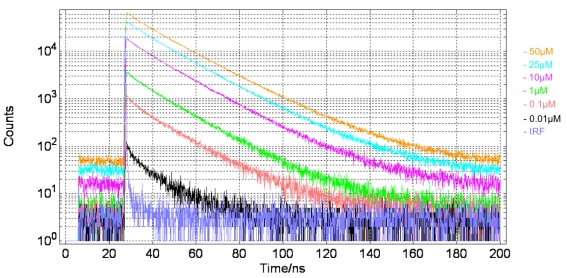
Time resolved decay curves of quantum dot sample, evaluated at different concentrations. Measurement made using external TCSPC hardware.

**Figure 14.**
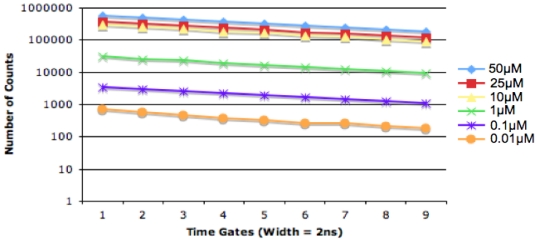
Fluorescence lifetime measurements, obtained from quantum dot samples using SPAD detector and on-chip circuitry.

A Nikon TE2000-U Microscope was used, with a ×20 objective and a PicoQuant 467 nm pulsed diode laser light source. The SPAD detector was placed at a side output port of the microscope. The IRF was obtained using a sample of Ludox to scatter the excitation light. An overview of the experimental setup is provided in Table 2. SPAD output pulses were processed using an external, commercially available TCSPC module (Becker and Hickl, SPC-130), Figure 13 and using the in-pixel, time-gated counter circuits, Figure 14.

The maximum number of counts in the peak channel of the decay curve increments appropriately according to the sample concentration and the decay curves remain parallel as they all represent the same sample lifetime. It was found that the SPADs were sensitive to approximately 0.01 μM. A reduction in the concentration of the quantum dot sample correlated closely with a reduction in the number of photon counts per second.

Table 3 below, summarizes the extracted lifetimes from the decay curves presented in Figures 12 and 13. Measurement error is based on 114 ps RMS SPAD jitter plus 4ps RMS TCSPC module jitter or 44 ps on-chip time-gate jitter. These lifetimes show how there is good agreement between the values captured using external TCSPC hardware and on-chip time-gated counters. The exception to this is the 0.01 μM sample, captured using TCSPC. At this low concentration only a small portion of the decay can be observed above the noise floor. This severely limits the fitting range that can be chosen for the lifetime extraction algorithm and can lead to skewed results.

**Figure 15.**
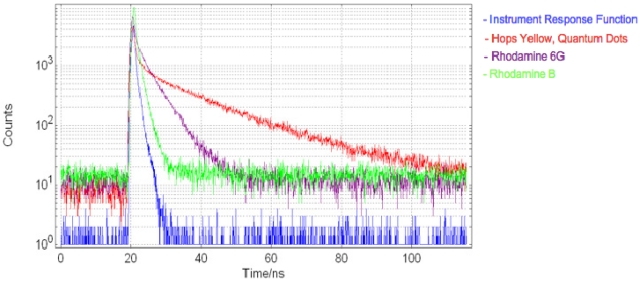
Fluorescence decay curves measured using two-chip micro-system, of quantum dot, Rhodamine 6G and Rhodamine B samples. An IRF of 910 ps FWHM is also included.

Measurements of fluorescence decay curves using the two-chip micro-system and external TCSPC hardware were obtained using quantum dots in a toluene solution (concentration = 57 μM) and Rhodamine 6G (concentration = 250 μM) and Rhodamine B (concentration = 100 μM) in water, Figure 15. Analysis of these decay curves yielded lifetime estimations of 13.81 ns, 4.36 ns and 1.34 ns for the quantum dot sample, Rhodamine 6G and Rhodamine B samples, respectively (±122 ps estimated measurement error, based on RMS SPAD and time-gate jitter). These results were performed with an LED excitation pulse width of 910 ps (FWHM) and using a sample volume of 45 μL. This is consistent with lifetimes reported in the literature [39,40]. Furthermore, quantum dot lifetimes are consistent with those measured using a conventional microscope system, confirming the ability of the micro-system to accurately resolve fluorescence lifetime data.

## 5. Conclusions

We have presented a micro-scale, CMOS-based single-photon sensitive detection system capable of sensing short lifetime fluorophores without lasers, PMTs or phoron counting acquisition cards. The limit of detection of the SPAD detector and in-pixel circuitry was found to be less than 10 nM and lifetimes could be captured with a resolution of 408 ps (minimum time-gate). The micro-LED driver is capable of producing optical pulses of 300 ps in width (FWHM) and a maximum DC optical output power of 550 μW.

We expect further improvements to this detection limit and acquisition time by inclusion of micro-optics to collimate the LED [41,42] and microlenses to recover detector fill factor [43]. Improved packaging to reduce vertical height between the chips and inclusion of microfluidic channels for sample delivery are necessary developments towards a complete, low-cost, portable chemical/bio-diagnostic device.

## Figures and Tables

**Table 1.** Summary of Micro-LED Driver Array.

Array Size	8 × 8
Driver Pitch	200 μm
Shortest Optical Pulse	777 ps
Excitation Wavelength	450 nm
Max. Voltage	5 V
Max. Driver Current	236 mA (DC)
VCO Frequency Range	7 MHz–800 MHz

**Table 2.** An overview of the experimental setup used in limiting dilution tests.

**Setting**	**Value**
Laser Rep. Rate	5 MHz
Laser Average Power	0.15 mW
Laser Emission Wavelength	467 nm
Microscope Objective	×20
SPAD Negative Bias	−19.5 V
SPAD Excess Bias	3.3 V
Sample	CdSe/ZnS Quantum Dots
Sample Volume	45 μL
Sample Emission Wavelength	548 nm

**Table 3.** Extracted lifetime values for quantum dot samples of varying concentration, processed using external TCSPC hardware and on-chip time-gated counters.

**Concentration**	**TCSPC Lifetime Value**	**On-Chip Time-Gated Lifetime Value**
50 μM	14.7ns (±114 ps)	13.7 ns (±122 ps)
25 μM	14.3 ns (±114 ps)	13.7 ns (±122 ps)
10 μM	14.2 ns (±114 ps)	13.5 ns (±122 ps)
1 μM	13.3 ns (±114 ps)	13.8 ns (±122 ps)
0.1 μM	14.6 ns (±114 ps)	13.1 ns (±122 ps)
0.01 μM	10.5 ns (±114 ps)	13.4 ns (±122 ps)
